# Novel *meso*-substituted porphyrin derivatives and its potential use in photodynamic therapy of cancer

**DOI:** 10.1186/s12885-021-08286-6

**Published:** 2021-05-13

**Authors:** Pablo Vallecorsa, Gabriela Di Venosa, M. Belén Ballatore, Dario Ferreyra, Leandro Mamone, Daniel Sáenz, Gustavo Calvo, Edgardo Durantini, Adriana Casas

**Affiliations:** 1 Centro de Investigaciones sobre Porfirinas y Porfirias (CIPYP), CONICET and Hospital de Clínicas Gral. José de San Martín, Universidad de Buenos Aires, Córdoba 2351 1er subsuelo, CP1120AAF Ciudad de Buenos Aires, Argentina; 2 IDAS-CONICET, Departamento de Química, FCEFQYN, Universidad Nacional de Río Cuarto, Río Cuarto, Córdoba, Argentina

**Keywords:** Photodynamic therapy, Porphyrins, Cancer, Photosensitisers, Cells, Mice

## Abstract

**Background:**

Photodynamic therapy (PDT) is an anticancer treatment that utilizes the interaction of light and a photosensitiser (PS), promoting tumour cell death mediated by generation of reactive oxygen species. In this study, we evaluated the in vitro photoactivity of four *meso*-substituted porphyrins and a porphyrin coupled to a fullerene.

**Methods:**

The cell line employed was the LM3 mammary adenocarcinoma, and the PS with the best photokilling activity was administered to mice bearing the LM3 subcutaneously implanted adenocarcinoma. The TEMCP^4+^ porphyrin and its analogue TEMCC^4+^ chlorine contain four identical carbazoyl substituents at the *meso* positions of the tetrapyrrolic macrocycle and have A_4_ symmetry. The TAPP derivative also has A_4_ symmetry, and it is substituted at the *meso* positions by aminopropoxy groups. The DAPP molecule has ABAB symmetry with aminopropoxy and the trifluoromethyl substituents in *trans* positions. The TCP-C_60_^4+^ dyad is formed by a porphyrin unit covalently attached to the fullerene C_60_.

**Results:**

The PSs are taken up by the cells with the following efficiency: TAPP> TEMCP^4+^ = TEMCC^4+^ > DAPP >TCP-C_60_^4+^, and the amount of intracellular PS correlates fairly with the photodamage degree, but also the quantum yields of singlet oxygen influence the PDT outcome. TAPP, DAPP, TEMCC^4+^ and TEMCP^4+^ exhibit high photoactivity against LM3 mammary carcinoma cells, being TAPP the most active. After topical application of TAPP on the skin of mice bearing LM3 tumours, the molecule is localized mainly in the stratum corneum, and at a lower extent in hair follicles and sebaceous glands. Systemic administration of TAPP produces a tumour: normal skin ratio of 31.4, and high accumulation in intestine and lung.

**Conclusion:**

The results suggest a potential use of topical TAPP for the treatment of actinic keratosis and skin adnexal neoplasms. In addition, selectivity for tumour tissue after systemic administration highlights the selectivity of and potentiality of TAPP as a new PS.

**Supplementary Information:**

The online version contains supplementary material available at 10.1186/s12885-021-08286-6.

## Background

Photodynamic therapy (PDT) is a technique that involves the use of a photosensitising substance (PS), which after being irradiated with a suitable wavelength in the presence of oxygen, undergoes photoactivation and reacts with cellular molecules promoting cell death mediated by generation of reactive oxygen species [1]. PDT can be used as an anticancer therapy mainly in dermatology and other malignancies, among other applications [2, 3]. It has been suggested that certain characteristics of tumours, as well as the vasculature, promote differential uptake of the PSs and therefore, the selective action of PDT [4].

First-generation PSs, such as Photofrin, exhibit poor clearance and lacks long-wavelength absorption. The synthesis of second-generation PSs moved towards modified tetrapyrrolic compounds, such as benzoporphyrin, chlorin and porphycene, which have a more intense long-wavelength absorption [5]. However, there is still an increasing need to develop new PSs showing better tumour selectivity, less cutaneous photosensitisation and increased photoactivity. Adequate PSs are expected to have a high absorption coefficient in the visible region of the spectrum and efficiently produce singlet oxygen [6].

The chemical structure of a PS plays a main role in the photosensitisation efficacy. An ideal PS has to be soluble in physiological media, and the degree of hydrophilicity and amphiphilicity directly influence the administration route, its biodistribution/pharmacokinetic profile and tumour selectivity [5].

Cationic porphyrins have several interesting features due to better solubilization in aqueous media and interaction with the cell membrane than their uncharged analogs, which make them attractive PSs. Besides, in recent years the *meso*-substituted porphyrins have gained interest in many areas of science and technology [7, 8].

Fullerenes have attracted considerable attention in different fields of science. Their unique carbon cage structure coupled with immense scope for derivatization make them a potential therapeutic agent. The fullerene family, especially C60, has unique photochemical and electrochemical properties that can be exploited [9].

Fullerene C_60_ linked to porphyrins show a higher capacity than porphyrins alone to form photoinduced processes [10]. Also, porphyrins connected to peripheral carbazoyl units represent efficient light-harvesting structures, where the carbazole groups can act as an antenna at lower wavelengths [11].

**Fig. 1:**
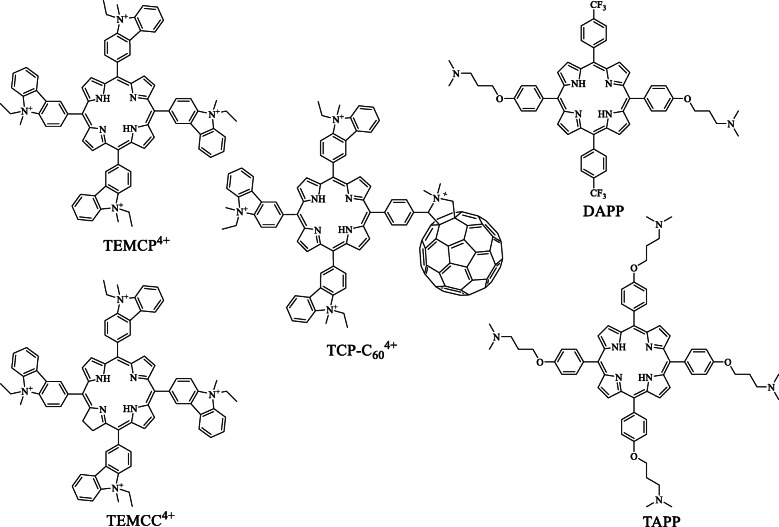
Molecular structures of the photosensitisers

In this study, different porphyrin derivatives were evaluated as potential phototherapeutic agents (Fig. 1). Both the TEMCP^4+^ porphyrin and its analogue TEMCC^4+^ chlorine show an A_4_ symmetry, with identical substituents at the *meso* positions of the tetrapyrrolic macrocycle. Thus, cationic carbazoyl groups provide four intrinsic charges at the periphery of the macrocycle [12]. The TAPP derivative has also A_4_ symmetry, however, unlike the previous porphyrins, it is substituted at the *meso* positions by aminopropoxy groups. These substituents contain an aliphatic chain that culminates with an amine group, which allows greater mobility and the basic groups can acquire positive charges by protonation in the cellular microenvironment. This effect can improve the interaction between the molecule and the cells [13, 14]. The DAPP molecule has ABAB symmetry with two substituent groups -the aminopropoxy and the trifluoromethyl groups- in *trans* positions. The amino groups, precursors of cations, can provide two positive charges that, in combination with the highly lipophilic trifluoromethyl groups, generate an amphiphilic structure [15]. Finally, the TCP-C_60_^4+^ dyad is formed by a porphyrin unit covalently attached to the fullerene C_60_. The resulting porphyrin has three identical carbazoyl substituents with positive charges, which together with the pyrrolidine cationic group attached to fullerene C_60_ provide four positive charges to the structure [16]. The synthesized compounds show the typical Soret absorption band 420–430 nm and four Q bands, between 515 and 650 nm, characteristic of derivatives of substituted tetraphenyl porphyrins. Besides, porphyrins can be converted to the corresponding chlorin derivatives, which are efficient photosensitizsers [12].

This work aimed to evaluate the in vitro photoactivity of four *meso*-substituted compounds (three porphyrins and one analogous chlorin) and a porphyrin coupled to a fullerene structure. The cell line employed was the LM3 murine adenocarcinoma, and the more active PS was selected to be administered in vivo in a model of mice bearing the subcutaneously implanted adenocarcinoma for the analysis of biodistribution and tumour selectivity after systemic administration and skin localization after topical application.

## Methods

### Chemicals

The synthetic *meso*-porphyrin derivatives: 5,10,15,20-tetrakis[4-(3-*N*,*N*-dimethylaminopropoxy) phenyl]porphyrin (TAPP), 5,15-di (4-[3-*N*,*N*-dimethylaminopropoxy]phenyl)-10,20-di(4-trifluoromethylphenyl) porphyrin (DAPP), 5,10,15,20-tetrakis[3-(*N*-ethyl-*N*-methylcarbazoyl)] porphyrin (TEMCP^4+^), 5,10,15,20-tetrakis[3-(*N*-ethyl-*N*-methylcarbazoyl)] chlorin (TEMCC^4+^) were synthesized as previously reported [12, 15]. The porphyrin-C_60_ dyad TCP-C_60_^4+^ was obtained as previously described [16] (Fig. 1 and Table 1). Further details on the synthesis of the compounds are shown in Supplementary material.

**Table 1:** Spectroscopic and photodynamic properties of the PSs in DMF

PS	ε^**soret a**^	λ_**abs**_^**b**^	λ_**em**_^**c**^	Φ_**F**_^**d**^	Φ_**Δ**_^**e**^	Reference
**TAPP**	1.64 × 10^5^	422	660	0.15	0.53	[17]
**DAPP**	1.67 × 10^5^	418	652	0.12	0.53	[15]
**TCP-C**_**60**_^**4+**^	2.49 × 10^5^	432	664	0.005	0.02	[16]
**TEMCC**^**4+**^	1.24 × 10^5^	432	656	0.23	0.49	[12]
**TEMCP**^**4+**^	2.80 × 10^5^	431	664	0.13	0.40	[12]

^a^ Molar absorption coefficient (L mol^− 1^ cm^− 1^)

^b^Maximum absorption wavelength of the Soret band

^c^Maximum absorption wavelength of the emission band

^d^Fluorescence quantum yield

^e^ Quantum yield of singlet oxygen production

Stock solutions of PSs dissolved in DMSO were kept at − 70 °C until use. They were dissolved in PBS at a maximum concentration of 0.1% DMSO for in vitro assays and in saline at 10% DMSO for in vivo studies.

MitoTracker® Green, LysoTracker® green and NBD-C6 ceramide were purchased from Thermo Fisher Scientific (Waltham, USA). MTT reagent was obtained from Sigma Aldrich Corp (St. Louis, USA).

All reagents and solvents were purchased from commercial suppliers and used without further purification.

### Cell lines

LM3 cells derived from the murine mammary adenocarcinoma M3 [18] were kindly provided by the Instituto Roffo, Buenos Aires, Argentina. These cells were previously characterized genetically and morphologically, and have not been retested and authenticated in the present study. They were routinely grown in RPMI-1640 medium containing 2 mM L-glutamine and phenol red, supplemented with 10% foetal bovine serum and incubated at 37 °C in an atmosphere containing 5% CO_2_. Cells were seeded at 7 × 10^4^ cells/ml and used 48 h after plating.

### PDT treatment

Cells were incubated in serum-free medium containing the PS (2.5 μM and 5 μM) and 3 h later, irradiations were performed. After irradiation, the cells were gently washed 3-times with PBS and the cells were incubated in medium supplemented with serum for another 19 h and then tested for viability. Light dose 50 (LD50) was calculated as the dose in mJ/cm^2^ necessary to induce 50% of cell death.

### MTT viability assay

Phototoxicity and cell viability were documented by the MTT assay [19]. This is a method based on the activity of mitochondrial dehydrogenases. Following appropriate treatments, MTT (3-[4,5-dimethylthiazol-2-yl]-2,5-diphenyltetrazoliumbromide) solution was added to each well in a concentration of 0.5 mg/ml, and plates were incubated at 37 °C for 1 h. The resulting formazan crystals were dissolved by the addition of DMSO and absorbance was read at 560 nm.

### Light source

A bank of 2 fluorescent lamps (Osram L 18 W/765) was used. The spectrum of light was between 400 and 700 nm with the highest radiant power at 600 nm. The 12-well plates containing the cells were located at a distance of 14 cm from the light source and were irradiated from below. The fluence rate was measured with a FieldMaster power meter and an LM3 HTP sensor (Coherent Inc., USA). We used fluences between 10 and 220 mJ/cm^2^ and the power density was 0.5 mW/cm^2^.

### Cellular uptake of PSs

The LM3 cells were exposed to different concentrations ranging from 0.15 to 5 μM of PSs in medium without serum during 3 or 24 h. The concentration ranges to perform intracellular PS accumulation profiles have been chosen based on MTT cytotoxicity assays (see Figure S1 and Table S1). After 3 consecutive washes with PBS, the PSs were extracted from the cells employing DMSO and afterwards, fluorescence was measured in a Perkin Elmer LS55 fluorimeter employing the maxima excitation and emission light wavelengths (Table 1). Standard solutions of PSs dissolved in DMSO were employed. The cellular uptake of the PSs per 10^5^ cells was determined from fluorescence calibration curves constructed by plotting the peak height of the PSs standard solutions. The calibration was linear within the employed range.

### Transport mechanism of TAPP into LM3 cells

To assess passive transport contribution, LM3 cells were: i) exposed to cold TAPP (1 μM and 5 μM) in serum-free medium 90 min at 4 °C; ii) exposed to pre-warmed TAPP in serum-free medium 90 min at 37 °C. After 3 consecutive washes with cold PBS, TAPP was extracted from the cells employing DMSO and quantified fluorimetrically as described in 2.6 employing TAPP standard solutions.

### Subcellular localization of TAPP

LM3 cells were plated into coverslips and 48 h afterwards, incubated in the dark during 5, 30 or 180 min at 37 °C with 5 μM TAPP and red fluorescence was microscopically documented. In addition, cells were exposed 180 min to 5 μM TAPP and then rinsed and incubated further 30 min with organelles specific fluorescent probes. To identify the Golgi apparatus, mitochondria and lysosomes, cells were labelled with 5 μM NBD-C6 ceramide, 0.1 μM MitoTracker® Green and LysoTracker® Green, respectively. Coverslips were *mounted* onto a glass microscope slide with *DABCO*–PBS mounting medium. Microscopic observation and photography were performed in an Olympus photomicroscope BX51.

Colocalization was quantified with Pearson’s correlation coefficient (PCC) employing Fiji image analysis software using the JaCOP plugin. Absolute PCC values of 1–0.7 indicate a relatively strong correlation, 0.69–0.36 a moderate correlation, 0.35–0.2 a weak correlation, and < 0.2 the absence of a correlation [20].

Fiji software [21] was also employed to quantify fluorescence intensity for the TAPP red channel. To measure fluorescence intensity of the images, RGB micrographs were splitted into grayscale images of the 3 channels. For TAPP red quantification, the green and blue channels were discarded. In order not to alter pixel values, the image was duplicated before processing. To accurately measure intensity values inside cell, the cell area was selected using the thresholding tools available, and when the region was properly bounded, it was saved as the region of interest using the ROI manager tool. This region fits accurately with the cellular area. Using the measure tool, both the whole area and the fluorescence of the selected region were quantified. Then, the fluorescence value inside the cell area was divided by the cell area.

### Animals

Male BALB/c mice 12 weeks old, weighing 20–25 g were used. The mice were purchased from the IMPAM Animal House, School of Medicine, University of Buenos Aires and housed in the Laboratory Animal Resources facility at the CIPYP during the experimental procedures. *Genotyping of mice w*as routinely carried out at the IMPAM Animal House. Healthy conventional mice were employed. They were provided with food (Purina 3, Molinos Río de la Plata, Argentina) and water ad libitum. Tumour inoculations were carried out after a period of acclimatization of 7 days. A suspension of 1.6 × 10^5^ cells of the LM3 cell line was subcutaneously injected on the left flank of mice. Animals were used at approximately day 20 after implantation. Tumor incidence was observed every two to 3 days for the 20-days period. Animals bearing tumours of the same uniform size were employed (1 cm diameter), and were divided into treatment and control groups by simple randomization. The sample size was calculated with G* power 3.1 software. Animals protocols were approved by the Argentinean Committee (CICUAL, School of Medicine, University of Buenos Aires) in full accord with the UK Guidelines for the Welfare of Animals in Experimental Neoplasia [22] and adhering to the ARRIVE guidelines.

### TAPP administration to mice

For topical and intraperitoneal administration, TAPP was dissolved in saline solution containing 10% DMSO immediately before use.

#### Topical administration

For topical application, 200 μl of the TAPP solution (100 μg TAPP) was applied on the tumour, after shaving the hair and rubbing with a paintbrush for 5 min (3 mice per experiment, 2 independent experiments). Fluorescence spectroscopy was performed on the skin over the tumour (SOT), and on the normal skin of the opposite flank, named ‘distant skin’. Skin sections were taken for fluorescence distribution imaging. Control animals (3 mice per experiment, 2 independent experiments) were topically treated with the vehicle. One week after TAPP treatment, skin biopsies of animals revealed no signs of photodamage.

##### In vivo fluorescence spectroscopy

In vivo fluorescence measurements were carried out for studying the kinetics of TAPP skin accumulation after topical application. A bifurcated fibre-optic probe was coupled to a Perkin-Elmer LS55 spectrofluorimeter, and fluorescence from the skin was detected at the probe tip. Fluorescence intensity was measured as a function of time and expressed in arbitrary units at an excitation wavelength of 420 nm and emission of 660 nm. In addition, fluorescence emission spectra were recorded to verify that the fluorescence signal corresponded to TAPP.

##### Fluorescence imaging of skin distribution following topical application of TAPP

SOT + tumour samples topically applied with TAPP, were frozen and 15-mm thick cryosections were prepared and handled under subdued lighting conditions. Microscopic observation was performed (Olympus BX51 microscope) using excitation at 545 nm using a bandpass filter and images were recorded using a Q-color 5 camera. After fluorescence observation, sections were stained with hematoxylin and eosin to identify structures of fluorescence localization.

#### Systemic administration of TAPP

For systemic administration, 150 μl (4 mg/kg mice) of TAPP was intraperitoneally injected to mice. Mice were sacrificed after 3, 24, 48 or 72 h after systemic administration (3 mice per time point). After determining the appropriate time point, the experiment was repeated twice (3 mice for TAPP treatment, 3 mice for controls). Before the sacrifice, mice were injected with heparin and afterwards, they were perfused with of sterile saline. Animals were euthanized with carbon dioxide. The organs samples were homogenized in DMSO, centrifuged for 30 min at 3000 g, and the supernatants were employed for the fluorimetric quantification of TAPP. A Perkin Elmer LS55 spectrofluorimeter (Buckinghamshire, UK) was used, employing excitation/emission wavelengths of 425/660 nm.

Control animals were injected with the vehicle. Mice were always kept in the dark until sacrifice.

Mice were carefully monitored daily. Under the above-explained conditions, the animals treated with TAPP did not show any visible signs of toxicity or significant change in body weight or food and water intake as compared to the controls. The biochemical indexes of liver and kidney function, as well as the blood counts, were not different from the controls (see supplementary material Tables S1, S2 and S3).

### Statistical treatment

The values in the figures and tables are expressed as means ± standard deviations of the mean. A two-tailed Student’s *t*-test with a preliminary normality test, was used to determine statistical significance between means. *P* values < 0.05 were considered significant. Simple linear regression analysis was performed using the GraphPad Software (version 6.0; GraphPad Prism).

## Results

### Cellular incorporation of PSs

**Fig. 2:**
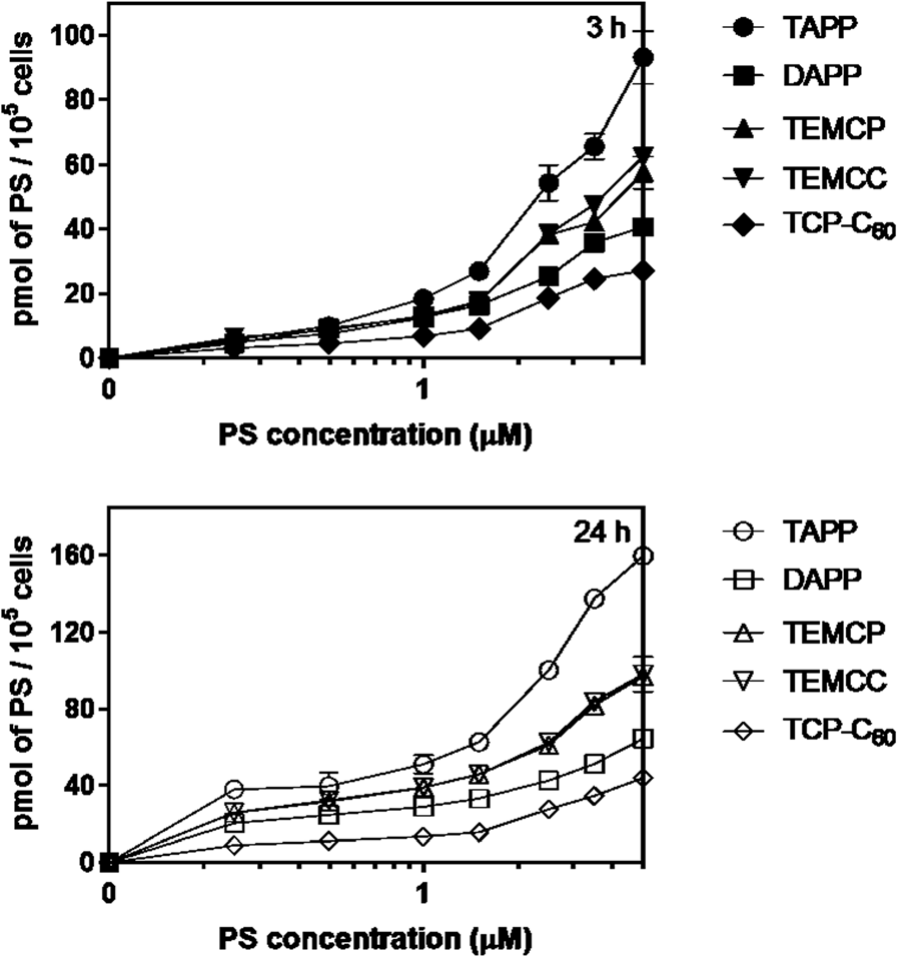
Cellular uptake of the photosensitiser as a function of the concentration. LM3 cells were exposed to different concentrations of the PSs during 3 h or 24 h. Intracellular photosensitiser levels were determined fluorimetrically and normalised per number of cells present at the end of the experiment. Error bars are the standard deviation for three independent experiments

LM3 cells were exposed to increasing non-dark toxic concentrations of the PSs during 3 h or 24 h (Fig. 2). Intracellular accumulated PSs increase linearly as a function of the concentration added at both periods (linear regression analysis, TAPP r^2^ = 0.9849; TEMCC^4+^ r^2^ = 0.9812; TEMCP^4+^ r^2^ = 0.9706; DAPP r^2^ = 0.9634; TCP-C_60_^4+^ r^2^ = 0.9547) for a 3 h period within the concentration range studied. This pattern is similar for all the PSs, with different slopes for each curve of incorporated PS vs concentration.

Uptake efficiency can be divided into 3 groups. For example, focusing in the 3 h- 5 μM profiles: a) TAPP, which is taken up by the cells with maximal efficiency (values higher than 60 pmol of PS/10^5^ cells); b) TEMCC^4+^, TEMCP^4+^and DAPP, being incorporated with medium efficacy (25 to 60 pmol of PS/10^5^ cells; and c) TCP-C_60_^4+^, which is poorly incorporated into the cells (less than 25 pmol of PS/10^5^ cells).

At 24 h, the values of incorporated PSs are higher than the ones obtained at 3 h, showing increases of 1.5 to 1.7-fold at the highest concentration employed.

### In vitro photodynamic therapy

**Fig. 3:**
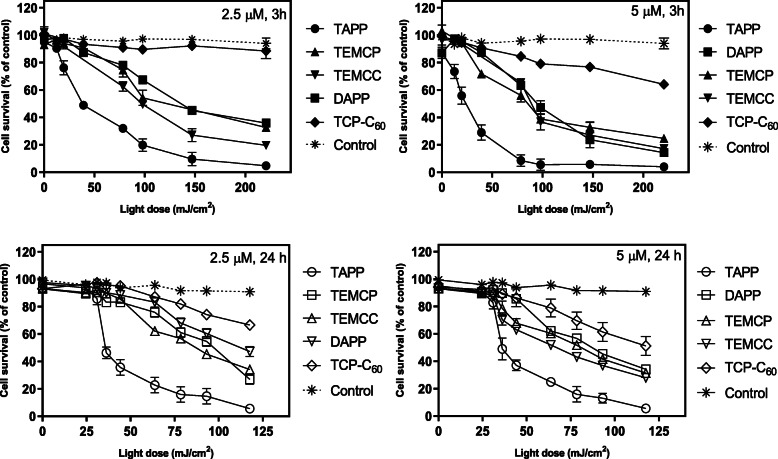
Cell survival after PDT. LM3 cells were incubated with 2.5 μM or 5 μM of the photosensitiser during 3 h or 24 h and afterwards, PDT was performed. Cell viability was evaluated by the MTT assay, as the percentage of photosensitiser-exposed non-irradiated cells. Control: cells exposed to the vehicle at the maximum concentration employed and light-treated. Error bars are the standard deviation for three independent experiments

PDT mediated by the action of the different PSs was studied as a function of the increasing light dose (Fig. 3). The conditions evaluated were 3 and 24 h incubation periods, and 2.5 and 5 μM concentrations. LD_50_s taken from Fig. 3 are summarised in Table 2 to help the analysis.

**Table 2:** LD_50_s of PDT mediated by the different PSs in LM3 cells, calculated from Fig. 2 values

LD_**50**_ (mJ/cm^**2**^)						
Incubation time	Concentration	TAPP	TEMCC^4+^	TEMCP^4+^	DAPP	TCP-C_**60**_^4+^
**3 h**	**2.5 μM**	35.7	100.6	101.2	136.2	> 220.0
	**5 μM**	22.9	88.9	92.0	99.8	> 220.0
**24 h**	**2.5 μM**	34.6	87.4	97.2	112.5	> 220.0
	**5 μM**	35.8	66.7	80.4	87.4	125.0

In line with the intracellular amount of incorporated PS, at 3 h of incubation, TAPP turned out to be the best PS, inducing LD_50_s of 35.7 and 22.9 mJ/cm^2^employing 2.5 μM y 5 μM respectively. TEMCC^4+^, TEMCP^4+^ and DAPP exert medium photosensitisation levels with LD_50_s between 100 and 136 mJ/cm^2^ at 2.5 μM, and a slightly higher photoactivity (89 to 100 mJ/cm^2^) at the highest concentration. TCP-C_60_^4+^was the least photoactive compound (LD_50_ > 220 mJ/cm^2^) at both concentrations.

Employing 24 h time exposures, LD_50_s are nor directly correlated with the intracellularly accumulated PS (see Fig. 2). As compared to 3 h, TAPP was equally photoactive at 2.5 μM and less photoactive at 5 μM, whereas TEMCC^4+^, TEMCP^4+^ and DAPP were 10 to 20% more active, and TCP-C_60_^4+^ increased remarkably its photosensitising efficacy, inducing an LD_50_ of 125 mJ/cm^2^. Considering the above-mentioned results, TAPP was considered the best photosensitizing molecule and was further studied. Figure S4 confirms that the damage observed after TAPP-PDT is due to reactive oxygen species generation.

### Transport mechanism of TAPP into LM3 cells

**Fig. 4:**
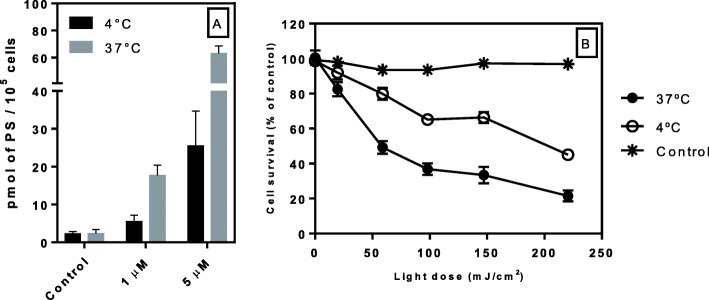
The transport mechanism of TAPP into LM3 cells. Cell uptake of TAPP at 1 μM and 5 μM at 4° and 37 °C during 90 min (**a**). Photodamage induced by the incorporated 5 μM TAPP for 90 min at 4° and 37 °C and illuminated at different light doses (**b**). Control: cells exposed to the vehicle and light-treated. Error bars are the standard deviation for three independent experiments

Figure 4 shows the contribution of passive transport of TAPP, inferred from its intracellular uptake and its differential photodynamic effect at 4 °C and 37 °C. An active transport system would be completely blocked by lowering the temperature to 4 °C, whereas the involvement of passive transport would imply uptake at low temperature [23]. Figure 4a shows that TAPP cellular uptake is impaired at 4 °C at about 60 to 70% as compared to the 37 °C conditions. In line with this result, LD_50_ increases from 68.0 mJ/cm^2^ to 202.5 mJ/cm^2^ by lowering the temperature, that is a 3-fold decline on the therapeutic efficacy (Fig. 4b).

### Fluorescence microscopy of cells exposed to TAPP

**Fig. 5:**
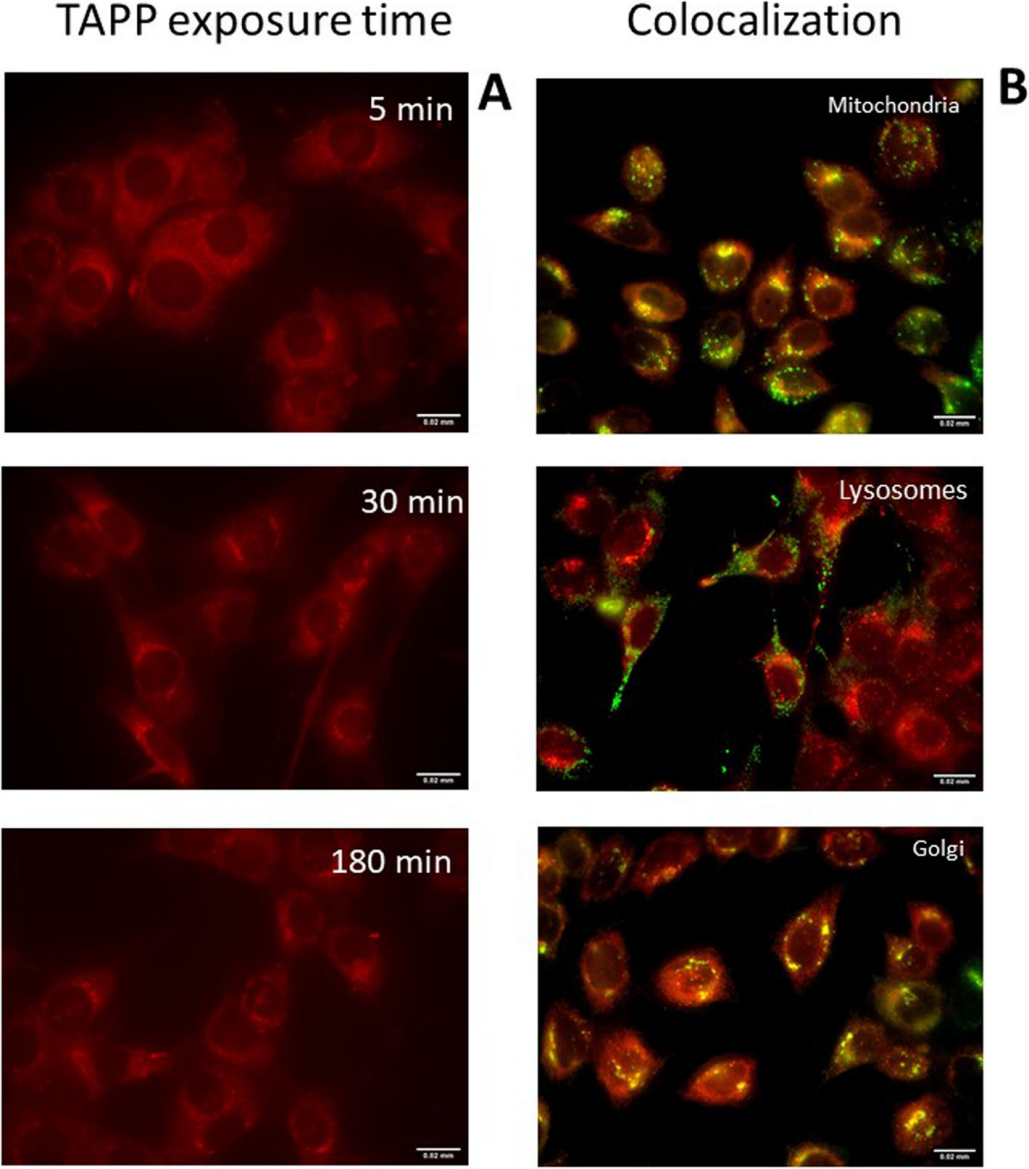
Intracellular TAPP fluorescence and colocalization. LM3 cells were exposed to 5 μM TAPP for 5 min, 30 min and 180 min, and red fluorescence was visualized by fluorescence microscopy (**a**). Cells exposed to TAPP for 180 min (red fluorescence) were then incubated with Lysotracker Green®, Mitotracker Green®, or NBD C6-Ceramide (green fluorescence) (**b**). Magnification 100X. These micrographs were representative of those obtained from three independent experiments

Fluorescence microscopy shows the time-dependence of TAPP cellular uptake at short incubation times. Intracellular TAPP fluorescence is observed at the 3 the time points analysed (Fig. 5a). After 5 min incubation, a diffuse cytoplasmic pattern can be distinguished, whereas, at 30 min and 3 h, a punctuate pattern becomes evidenced, suggesting confinement to subcellular organelles. The quantification of red fluorescence indicates that equal fluorescence values are attained at the 3 time points (data not depicted).

In Fig. 5b we studied the colocalization of TAPP after 3 h exposure with organelle-specific probes. PCC values quantifying the colocalization of the organelles fluorescent marker and TAPP are as follows: mitochondria: 0.36 ± 0.08, lysosomes: 0.36 ± 0.05 and Golgi apparatus: 0.68 ± 0.03. The results suggest that TAPP mainly localizes in the Golgi apparatus and at a lesser extent in mitochondria and lysosomes. In addition, a diffuse cytoplasmatic signal, not ascribable to organelles can be distinguished.

### In vivo studies

#### TAPP topical application

**Fig. 6:**
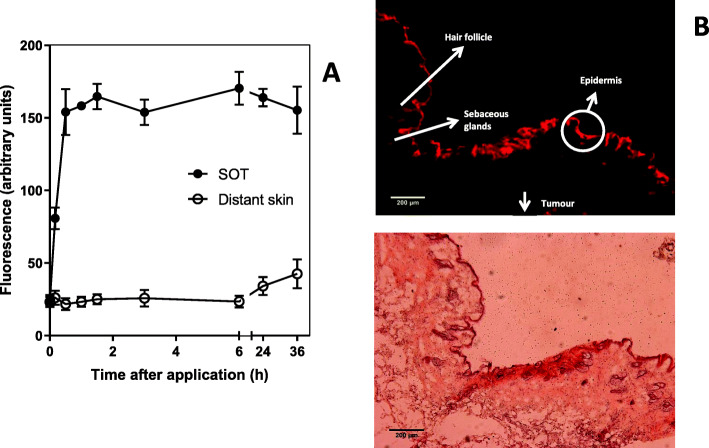
TAPP accumulation in the skin over the tumour (SOT) and distant skin after topical application of TAPP on the SOT of LM3 bearing mice. TAPP was applied topically on SOT (100 μg TAPP in saline containing 10% DMSO). Animals were kept in the dark. At different times after application, TAPP fluorescence was monitored. Determinations were made on SOT and over a distant skin area. The average of three mice per treatment in 2 independent experiments is shown (**a**). Fluorescence and hematoxylin and eosin-stained images of SOT after 24 h of TAPP application. Magnification 10X objective (**b**)

Figure 6 shows TAPP accumulation in SOT and distant skin after topical application on LM3 tumours. SOT: distant skin fluorescence ratio is around 7 between 30 min and 6 h after application and around 4 at 36 h, showing TAPP retention in the application site.

To analyse the penetration depth of TAPP through the skin, its fluorescence localization was observed microscopically in SOT after topical application of TAPP. TAPP is mainly retained in the epidermis but does not further diffuse to the dermis, subcutaneous adipose tissue or the subcutaneous-located tumour. In hair follicles and sebaceous glands, TAPP fluorescence is also observed. The H&E staining reveals the absence of damage to the skin induced by TAPP application in mice kept in the dark.

#### Systemic TAPP biodistribution

A non-toxic amount of TAPP (4 mg/kg) was injected intraperitoneally to tumour-bearing mice. Three, 24, 48 or 72 h after TAPP systemic administration, the amount of TAPP was quantified in the tumour and the organs. Maximal accumulation of TAPP was observed in all the tissues at 24 h post-administration. However, even at 72 h afterwards, fluorescence was still present in tissues such as tumour and SOT (data not shown).

**Fig. 7:**
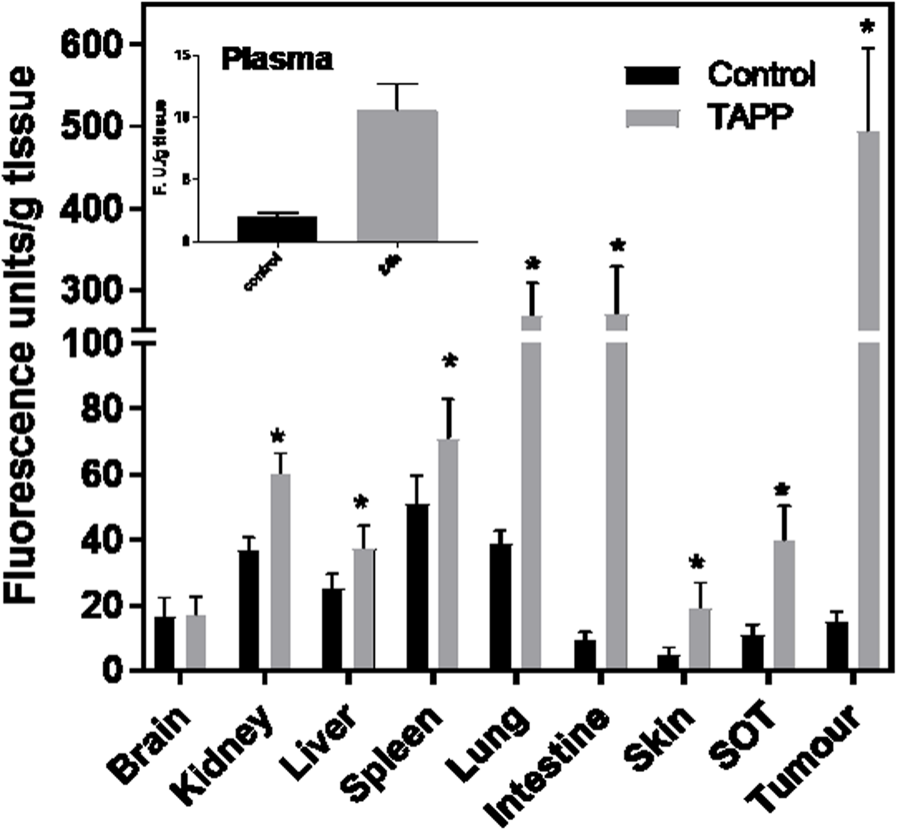
Biodistribution of TAPP after systemic administration. TAPP was intraperitoneally administrated to LM3 tumour bearing mice (4 mg/kg) and after 24 h of administration, TAPP was extracted from tissues and quantified. The results are expressed as fluorescence units/g tissue (means ± SD). The average of three data in 2 independent experiments is shown. ^*^ *P* < 0.01 as compared to the control injected with the vehicle (two-tailed Student’s *t*-test)

Figure 7 depicts the accumulation of TAPP obtained at 24 h after i.p. injection. Basal autofluorescence values varied among tissues, and for that reason, autofluorescence of control tissues is also depicted. The amount of TAPP is maximal in tumour tissue. Besides, SOT accumulates significantly more TAPP than normal skin (*p* = 0.004, Student’s *t*-test). These values lead to a high selectivity index of 13 for tumour: SOT ratio and 26 for tumour: normal skin.

Besides, elevated TAPP concentrations were registered in the lung and intestine. On the other hand, lower values of TAPP were found in the rest of the organs such as distant skin, kidney, liver and spleen. It is quite important to remark that the brain did not accumulate TAPP at all. Plasmatic TAPP was detected up to 72 h after administration (data not shown).

## Discussion

In the present work, we have found that TAPP, DAPP, TEMCC^4+^ and TEMCP^4+^ exhibit high photoactivity against LM3 mammary carcinoma cells. Among them, TAPP was the most active molecule, and its biodistribution was tested after topical application and systemic administration.

Previous experiments showed that TEMCC^4+^ was a highly effective agent to photo-induce the hemolysis of human red blood cells, whereas TEMCP^4+^ induced hemolysis at a lower extent due to haemoglobin absorption [12]. Furthermore, TAPP demonstrated to be an efficient generator of singlet oxygen in the presence of oxidizable substrates, such as 9,10-dimetylanthracene and L-tryptophan in biomimetic media [17]. On the other hand, porphyrin-fullerene C_60_ dyads with high ability to form photoinduced charge-separated state were evaluated as PS with potential application in the photoinactivation of *S. aureus* [16].

Cellular uptake of the PSs employed in the present work suggests that all the PSs assayed are incorporated linearly as a function of the added concentration, suggesting a major involvement of passive transport in the uptake of the PSs. The different slopes of the curves of incorporated PS as a function of the concentration suggest different incorporation rates.

Our results suggest that at 3 h of exposure, the amount of intracellular PS correlates fairly with the photodamage degree. However, even when at 24 h the intracellular accumulated PS is higher than at 3 h, a direct correlation with the photodamage degree was not always observed. Whereas TEMCC^4+^, TEMCP^4+^ and DAPP were 10 to 20% more photoactive at 24 h as compared to 3 h, TAPP employed at 5 μM concentration, was less photoactive at longer incubation times and TCP-C_60_^4+^ was the only PS that increased remarkably its efficiency of photosensitisation.

The different behaviour for each PS suggests that depending on the molecule and the intracellular concentration, the photoactivity is variable. The different substituents, as well as the degree of axial symmetry, have been suggested to contribute to the cellular uptake. The positive charges located in the molecule periphery, increased the amphipathic character of the PS, thus contributing to the interaction with cell membranes [7]. In the present study, TAPP is taken up by the cells at a higher rate than the rest of the PSs.

TAPP bears four mobile dimetilaminopropyl groups as precursors of cationic centres. Considering the pK_a_ values of these substituents [24], a high percentage of the amine groups can acquire positive charges by protonation in water, favouring a better interaction with cells. In the cases of TEMCP^4+^ and TEMCC^4+^, although these porphyrin derivatives have four carbazoyl substituents with intrinsic cationic groups, the cationic groups are in a rigid macrocycle structure and less exposed to interact with cells. On the other hand, DAPP has only two amine groups, while the dyad TCP-C_60_^4+^ contains four rigid charges and a bulky and highly lipophilic C_60_ group that decreases an effective interaction with the cells.

Besides, TAPP, DAPP, and TEMCC4+ have quantum yields of singlet oxygen production in the range of 0.49 to 0.53, and TEMCP4+ has a slightly lower (0.40) quantum yield.

In terms of efficiency of photosensitization, it is worth to note that after 3 h of incubation, TAPP induced on LM3 cells an LD_50_s of 35.7 and 22.9 mJ/cm^2^ employing 2.5 μM and 5 μM concentrations, respectively. On the other hand, after 3 h exposure of the same cell line to the already marketed pro-photosensitizer 5-aminolevulinic acid (25 μM), it was previously reported by us that a light dose of 150 mJ/cm^2^ (same light array) was required to induce the same level of cell killing [25]. This comparison suggests that the porphyrin TAPP is a promising PS.

The impaired action of 5 μM TAPP at longer incubation times may be ascribed to saturation of the intracellular targets and, at higher concentrations, TAPP molecules could be aggregated, thus inducing a diminished activity. On the other hand, the higher action of the porphyrin coupled to the fullerene at long incubation periods may suggest better solubilization as monomers since this structure is prone to form aggregates in aqueous media, increasing the photodynamic activity. Besides, the action of proteases may be needed to act at long times of exposure to release the porphyrin from the C_60_ structure.

TCP-C_60_^4+^ is the least photoactive porphyrin, and this is correlated with a poor cellular uptake and, in addition to a low singlet oxygen quantum yield in polar media, which is 6-fold lower as compared to the other PSs. The poorer uptake of the molecule is probably due to the presence of a voluminous group and the scarce endocytic ability of the LM3 cells. In addition, as suggested previously [26], drugs coupled to fullerenes can significantly differ from the free drug in terms of cellular uptake and subcellular distribution.

A major contribution of passive transport in the cellular uptake of TAPP is line with its reported Log P of 1.67 [27]. Besides, Golgi major localization, as well as moderate localization in lysosomes and mitochondria and diffuse cytoplasmatic pattern, is observed at 3 h of TAPP exposure. Lysosomes and the trans-Golgi network usually have an acidic lumen with pH of approximately 5 and 6.5, respectively, so that weak bases are likely to be charged at a higher extent [28], thus exhibiting more marked fluorescence. The subcellular location of a PS is known to have a strong influence on the cell death response to photoinactivation .

Among the 5 analysed molecules, TAPP was the most photoactive in the cell line employed, and thus, was the chosen PS to be employed in a tumour bearing mice model. After topical application, TAPP was almost completely retained in the stratum corneum of the skin, without diffusion to the deeper layers of the skin. However, some fluorescence can be distinguished in the hair follicles and sebaceous glands suggesting a contribution of the transfollicular route of drug delivery. Specific localization of TAPP in the stratum corneum suggests the use of TAPP-PDT for the treatment of actinic keratosis.

As well, recent reports demonstrated that the transepidermal route, hair follicles, and sebaceous glands significantly contribute to topical or transdermal delivery. Malignant cutaneous adnexal neoplasms form a group of rare, typically low-grade-malignancy carcinomas with follicular, sebaceous, apocrine, or eccrine differentiation or a combination of the first 3 subtypes, and PDT has already been performed in some of these diseases [29, 30]. The presence of TAPP in hair follicles and sebaceous glands, suggests the possibility to use this molecule in the photodynamic treatment of skin adnexal neoplasms.

Systemic administration of TAPP produce accumulation of high quantities of TAPP in tumour and SOT (tumour invaded skin), showing a high tumour: normal skin ratio of 31.4. It is also interesting to note the lack of accumulation of TAPP in brain tissue, evidencing that the molecule does not passage through the blood-brain barrier. Besides, high intestine and lung TAPP values suggest the possibility of employing TAPP-PDT for the treatment of colon and lung cancer, since both organs are easily accessible by optic fibres if further studies confirm the accumulation of TAPP in tumours of that origin.

The key conclusion of this study was that TAPP was the PS that showed the best performance in vitro. Upon topical application to tumour bearing mice, TAPP was retained in the stratum corneum and adnexal glands, having a potentiality in the treatment of superficial malignancies or pre-malignancies such as actinic keratosis and skin adnexal neoplasms after topical application. After systemic administration, it is highly selective for tumour tissue, thus having promising uses in PDT treatment of non-dermatologic conditions.

## Supplementary Information

**Additional file 1.**

## Data Availability

All of the data supporting our findings can be found in the main paper and Supplementary material. Data are however available from the authors upon reasonable request.

## Abbreviations

DAPP: 5,15-di (4-[3-*N*,*N*-dimethylaminopropoxy]phenyl)-10,20-di (4-trifluoromethylphenyl) porphyrin
MTT: 3-[4,5-dimethylthiazol-2-yl]-2,5-diphenyltetrazoliumbromide)
PCC: Pearson’s correlation coefficient
PDT: Photodynamic therapy
PS: Photosensitizer
SOT: Skin over the tumour
TAPP: 5,10,15,20-tetrakis[4-(3-*N*,*N*-dimethylaminopropoxy) phenyl]porphyrin
TEMCC^4+^: 5,10,15,20-tetrakis[3-(*N*-ethyl-*N*-methylcarbazoyl)] chlorin (TEMCC^4+^)
TEMCP^4+^: 5,10,15,20-tetrakis[3-(*N*-ethyl-*N*-methylcarbazoyl)] porphyrin
