# Supplementary material for: Novel meso-substituted porphyrin derivatives and its potential use in photodynamic therapy of cancer
Source: BMC Cancer. 2021 May 13;21:547. doi: 10.1186/s12885-021-08286-6 (PMC8117271; doi:10.1186/s12885-021-08286-6)
Supplement: Supplementary file 1 — Additional file 1. [file 12885_2021_8286_MOESM1_ESM.docx]

**Supporting Information**

**Novel meso-substituted porphyrin derivatives and its potential use in photodynamic therapy of cancer**

Pablo Vallecorsa^a^, Gabriela Di Venosa^a^, M. Belén Ballatore^b^, Dario Ferreyra^b^, Leandro Mamone^a^, Daniel Sáenz^a^, Gustavo Calvo^a^, Edgardo Durantini^b^ & Adriana Casas^a^

^a^Centro de Investigaciones sobre Porfirinas y Porfirias (CIPYP). CONICET-Hospital de Clínicas Gral. José de San Martín, Ciudad Autónoma de Buenos Aires, Argentina.

^b^IDAS-CONICET, Departamento de Química, FCEFQYN, Universidad Nacional de Río Cuarto, Río Cuarto, Córdoba, Argentina.

**Corresponding author**

Dr. Adriana Casas

E-mail: adriana@qb.fcen.uba.ar

**Table of Contents**

**1. Chemical synthesis** Page S2

**1.1 Materials** Page S2

**1.2 Synthesis of porphyrin derivatives** Page S2

**2. Biological assays** Page S5

**2.1 Toxicity assays in non-tumor-bearing mice** Page S5

**2.2 PSs dark toxicity on cells** Page S7

**2.3 ROS production in LM3 cells after TAPP-PDT**  Page S8

**3. References** Page S9

**1.Chemical synthesis**

**1. 1 Materials**

Chemicals were obtained from Sigma-Aldrich (Milwaukee, WI, USA). They were used without further purification. Organic solvents (GR grade) from Merck (Darmstadt, Germany) were distilled and maintained on molecular sieves. Silica gel thin-layer chromatography (TLC) plates 250 microns from Analtech (Newark, DE, USA) were used. Ultrapure water was obtained from a Labconco (Kansas, MO, USA) equipment model 90901-01.

**1.2 Synthesis of porphyrin derivatives**

**5,10,15,20-tetrakis[4-(3-*N*,*N*-dimethylaminopropoxy) phenyl]porphyrin (TAPP, Scheme S1)**. A solution of 4-(3-*N*,*N*-dimethylaminopropoxy)benzaldehyde (151 μL, 0.75 mmol) and *meso*-[4-(3-*N*,*N*-dimethylaminopropoxy)phenyl]dipyrromethane (242 mg, 0.75 mmol) in 80 mL of dichloromethane was purged with argon for 15 min. Then, trifluoroacetic acid (TFA, 255 μL, 3.3 mmol) was added and the solution was stirred for 30 min at room temperature. After that, the mixture was treated with TEA (450 μL, 3.2 mmol) and the volatile compounds were removed under reduced pressure. The solid was re-dissolved in 250 mL of dichloromethane. Then, 2,3-dichloro-5,6-dicyano-1,4-benzoquinone (DDQ, 367 mg, 1.6 mmol) was added and the mixture was stirred for an additional 3 h at reflux. The solvent was removed under vacuum and flash column chromatography (silica gel, dichloromethane/methanol 10-20 % gradient/ TEA 0-3 % gradient) yielded 25 mg (6.3 %) of TAPP. Spectroscopic data agree with those previously reported [1].

**Scheme S1**. Synthesis of TAPP. Reagents and conditions: (a) TFA, Ar, 30 min; (b) TEA; (c) DDQ.

**5,15-di(4-[3-*N*,*N*-dimethylaminopropoxy]phenyl)-10,20-di(4-trifluoromethylphenyl) porphyrin (DAPP, Scheme S2)**. A solution of 4-(trifluoromethyl)benzaldehyde (0.80 g, 4.7 mmol) and *meso*-[4-(3-*N*,*N*-dimethylaminopropoxy)phenyl]dipyrromethane (1.28 g, 4.0 mmol) in 400 mL of dichloromethane was purged with argon for 15 min. Then, TFA (1.23 mL, 16.0 mmol) was added and the solution was stirred for 30 min at room temperature. The mixture was treated with TEA (2.1 mL, 15 mmol) and the solvents were removed under reduced pressure. The solid was re-dissolved in 250 mL of dichloromethane and refluxed for 3 h in the presence of DDQ (1.82 g, 8 mmol). The solvent was removed under vacuum and flash column chromatography (silica gel, dichloromethane/methanol 7-20 % gradient/ TEA 2 %) afforded 90 mg (4.7 %) of the pure DAPP. Spectroscopic data agree with those previously reported [1].

**Scheme S2**. Synthesis of DAPP. Reagents and conditions: (a) TFA, Ar, 30 min; (b) TEA; (c) DDQ.

**5,10,15,20-tetrakis[3-(*N*-ethyl-*N*-methylcarbazoyl)]chlorin (TEMCC^4+^, Scheme S3)**. This chlorin was obtained from 5,10,15,20-tetrakis[3-(*N*-ethylcarbazoyl)]chlorin (TECC), which was synthesized as previously described [2]. A solution of TECC (30 mg, 0.030 mmol) and dimethyl sulphate (1.75 mL) was heated to 90 ºC with stirring under argon atmosphere for 24 h. The mixture was cooled and the product was precipitated with ether. The solid was separated by centrifugation and washed with ether, Na_2_CO_3_ (2% w/v) and water yielding 39 mg (95%) of TEMCC^4+^. Spectroscopic data agree with those previously reported [2].

**Scheme S3**. Synthesis of TEMCC^4+^. Reagents and conditions: (a) (CH_3_)_2_SO_4_, 90 ºC, 24 h.

**5,10,15,20-tetrakis[3-(*N*-ethyl-*N*-methylcarbazoyl)]porphyrin (TEMCP^4+^, Scheme S4)**. This porphyrin was synthesized from 5,10,15,20-tetrakis[3-(*N*-ethylcarbazoyl)]porphyrin (TECP) that was obtained as previously designated [2]. Methylation of TECP was performed as described above for TEMCC^4+^, using TECP (15 mg, 0.014 mmol) to obtain 18 mg (97%) of TEMCP^4+^. Spectroscopic data agree with those previously reported [2].

**Scheme S4**. Synthesis of TEMCP^4+^. Reagents and conditions: (a) (CH_3_)_2_SO_4_, 90 ºC, 24 h.

**Porphyrin-C_60_ dyad (TCP-C_60_^4+^, Scheme S5)**. 5-(4-Formylphenyl)-10,15,20-tris[3-(*N*-ethylcarbazoyl)]porphyrin was synthesized as previously described [3]. A solution of fullerene C_60_ (158 mg, 0.22 mmol), 5-(4-formylphenyl)-10,15,20-tris[3-(*N*-ethylcarbazoyl)]porphyrin (212 mg, 0.21 mmol) and *N*-methylglycine (37 mg, 0.42 mmol) in 150 mL of dry toluene was stirred at reflux in atmosphere of argon for 18 h. Then, the solvent was removed under vacuum. Flash column chromatography (silica gel) using toluene as eluent afforded 230 mg (62%) of TCP-C_60_. Then, a mixture of TCP-C_60_ (10 mg, 6x10^-3^ mmol) and dimethyl sulfate (2 mL, 21.10 mmol) was stirred for 44 h at 90 ºC. The methylated dyad was precipitated with ethyl ether and the solid was washed with 5% aqueous Na_2_CO_3_ and then with water to obtain 96% of TCP-C_60_^4+^. Spectroscopic data agree with those previously reported [3].

**Scheme S5**. Synthesis of TEMCP^4+^. Reagents and conditions: (a) toluene, Ar, reflux, 18 h; (b) (CH_3_)_2_SO_4_, 90 ºC, 44 h.

**2. Biological assays**

**2.1 Toxicity assays in non-tumour-bearing mice**

After topical application of 200 µl of the TAPP solution (100 µg TAPP) on the shaved normal skin of BALB/c mice, the animals were exposed to 12 h dark/light cycles (300 lux in the room at cage level). One week after TAPP treatment, skin biopsies of animals revealed no signs of photodamage.

The dose of TAPP employed for systemic administration to normal mice was chosen according to the maximal solubility of the compound in the vehicle. Four mice were injected with TAPP and 4 with the vehicle.

The mice weights were recorded before and daily after TAPP administration for one week, time at which mice sacrificed. Animals were also monitored for behavioural, neurological and autonomic parameters, and the food and water intake were recorded daily throughout the experiment (Table S1). Previous to sacrifice, animals were anaesthetized, and blood samples were collected from the mice’s eyes and serum was harvested by centrifugation. Then, all the mice were sacrificed and haemogram, ALT, AST and ALP, blood urea nitrogen (BUN), uric acid and creatinine were determined (Tables S2 and S3).

Under the above explained conditions, the animals treated with TAPP did not show any visible signs of toxicity, or significant change in body weights or food and water intake as compared to the controls. The biochemical indexes of liver and kidney function as well as the blood counts were not different than the controls.

**Table S1: Effects of TAPP on physical parameters of mice**

|  | Day 0 | | Day 7 | |
| --- | --- | --- | --- | --- |
|  | Control | TAPP-PDT | Control | TAPP-PDT |
| Body weight (g) | 24.2±1.3 | 25.7±1.1 | 24.1±1.2 | 26.2±1.4 |
| Food intake/day (g) | 3.2±0.3 | 3.0±0.3 | 3.3±0.4 | 3.1±0.3 |
| Water intake/day (ml) | 4.1±0.4 | 4.0±0.2 | 3.9±0.4 | 4.1±0.3 |

**Table S2: Effect of TAPP on blood parameters**

|  | Control | TAPP |
| --- | --- | --- |
| ALT (IU/L) | 58±4 | 50±6 |
| AST (IU/L) | 135±12 | 141±15 |
| ALP (IU/L) | 178±20 | 184±16 |
| BUN (mg/dL) | 20.5±3.1 | 18.7±1.9 |
| Uric acid (mg/dL) | 0.8±0.05 | 0.7±0.08 |
| Creatinin (mg/dL) | 0.4±0.03 | 0.4±0.02 |

**Table S3: Effect of TAPP on haemogram**

|  | Control | TAPP |
| --- | --- | --- |
| Hemoglobin (g/dL) | 13.1±1.4 | 14.5±1.6 |
| RBC (10^6^/mm^3^) | 6.7±0.8 | 7.1±0.9 |
| Hematocrit (%) | 45.3±5.2 | 48.3±5.2 |
| MCV (fL) | 54.5±6.1 | 52.2±6.1 |
| MCHC (g/dL) | 31.3±3.8 | 30.2±4.2 |
| Platelets (10^3^/mm^3^) | 450±53 | 543±61 |
| Total leukocytes (10^3^/mm^3^) | 6.6±0.7 | 6.7±0  .3 |
| NEUT (%) | 22.5±3.4 | 26.3±2.8 |
| LYMP (%) | 70.3±6.1 | 65.4±7.1 |
| EOS (%) | 1.1±0.3 | 1.6±0.3 |
| MONO (%) | 6.5±0.8 | 7.8±0.9 |

**2.2 Dark toxicity of PSs on cells**

**Figure S1: Dark toxicity of PSs.** LM3 cells were incubated with increasing concentrations of the PSs during 3 h (A) or 24 h (B) and 4 h afterwards, cell viability was evaluated by the MTT assay, as the percentage of the cells exposed to the vehicle.

**Table S4: IC50s for LM3 cells (µM)**

|  | **TAPP** | **DAPP** | **TEMCP** | **TEMCC** | **TCP-C_60_** |
| --- | --- | --- | --- | --- | --- |
| **3 h** | 52.4±4.8 | 59.0±7.8 | 54.2±5.3 | 58.0±4.2 | 46.4±4.9 |
| **24 h** | 56.2±5.3 | 54.01±7.9 | 61.5±8.1 | 56.6±6.8 | 35.7±4.0 |

**2.3 ROS production in LM3 cells after TAPP-PDT**

PDT-induced cell death occurs through generation of intracellular ROS. ROS production was quantified using a cellular reactive oxygen species detection assay which uses the cell permeant reagent 2’,7’ –dichlorofluorescin diacetate (DCFDA, Sigma Chem Co, USA). DCFDA is a fluorogenic dye that measures hydroxyl, peroxyl and other ROS within the cells [4]. Fig. S2 indicates that after cell treatment with PDT mediated by TAPP, the amount of ROS is significantly elevated.

**
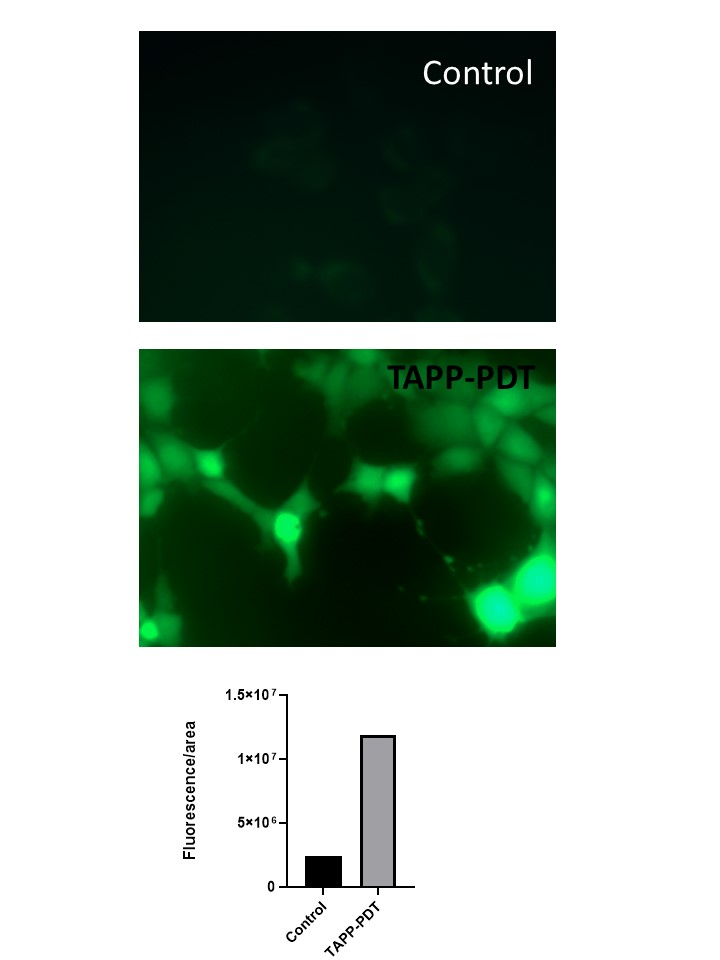
**

**Fig. S2: ROS production in LM3 cells**

LM3 cells were grown on coverslips, incubated 3 h with 5 µM TAPP and illuminated with 30 mJ/cm^2^. Afterwards, they were exposed to 10 µM DCFDA for 30 min at 37 °C and after washing, fluorescein was visualized in an epifluorescence microscope. Non-illuminated controls were performed. Magnification 100X. Quantification of fluorescence intensity is depicted.

**3. References**

1. Caminos DA, Durantini EN (2005) Synthesis of asymmetrically meso-substituted porphyrins bearing amino groups as potential cationic photodynamic agents. J Porphyr Phthalocyanines. https://doi.org/10.1142/S1088424605000423

2. Ferreyra DD, Spesia MB, Milanesio ME, Durantini EN (2014) Synthesis and photodynamic properties of 5,10,15,20-tetrakis[3-(N-ethyl-N- methylcarbazoyl)]chlorin and its analogous porphyrin in solution and in human red blood cells. J Photochem Photobiol A Chem 282:16–24. https://doi.org/10.1016/j.jphotochem.2014.02.004

3. Ballatore MB, Spesia MB, Milanesio ME, Durantini EN (2014) Synthesis, spectroscopic properties and photodynamic activity of porphyrin-fullerene C60 dyads with application in the photodynamic inactivation of Staphylococcus aureus. Eur J Med Chem. https://doi.org/10.1016/j.ejmech.2014.06.077

4. Wu D, Yotnda P (2011) Production and detection of reactive oxygen species (ROS) in cancers. J Vis Exp. https://doi.org/10.3791/3357
